# Study of biofilm formation and antibiotic resistance pattern of gram-negative Bacilli among the clinical isolates at BPKIHS, Dharan

**DOI:** 10.1186/s13104-019-4084-8

**Published:** 2019-01-18

**Authors:** Rabina Dumaru, Ratna Baral, Lok Bahadur Shrestha

**Affiliations:** Microbiology & Infectious Diseases, B.P Koirala Institute of Health Sciences, Dharan, 56700 Nepal

**Keywords:** Gram-negative bacilli, Extended-spectrum beta-lactamases, Metallo-beta-lactamases, Biofilm, Multi-drug resistant

## Abstract

**Objectives:**

Gram-negative bacilli are the common causative agents for community-acquired, nosocomial and opportunistic infections. The recent upsurge of biofilm, as well as beta-lactamases producing strains, have synergistically led to the extensive dissemination of multi-drug resistant gram-negative bacilli. This study was carried out with an intention to detect the biofilm formation by gram-negative bacilli and determine their antibiogram along with the detection of extended-spectrum beta-lactamases (ESBLs) and metallo-beta-lactamases (MBLs) production.

**Results:**

Among 314 isolates, *Escherichia coli* (38%) were the predominant isolates followed by *Acinetobacter* spp. (20%), *Klebsiella* spp. (16%), and *Pseudomonas* spp. (12%). Overall, 197 (62.73%) of isolates were biofilm positive. 84 (26.75%) and 51 (16.24%) were confirmed as ESBL and MBL producers respectively. The association between MBL production and biofilm formation was statistically significant *(*χ^2^ = 10.20, *P* value= 0.002) whereas it was insignificant between ESBL and biofilm production (χ^2^ = 0.006, *P*-value= 0.937). Most of the biofilm and MBL producing strains were multi-drug resistant.

## Introduction

Gram-negative bacilli, most commonly *Escherichia coli*, *Klebsiella pneumoniae*, *Pseudomonas aeruginosa*, and *Acinetobacter baumannii* are responsible for causing various pathological diseases like UTIs, septicemia, pneumonia, nosocomial infections, opportunistic infections etc. [1]. Beta-lactams were wonder drugs until the dissemination of beta-lactamases (ESBL and MBL) producing strains were detected. “extended-spectrum beta-lactamases (ESBLs) may be defined as plasmid-mediated enzymes that hydrolyze oxyimino-cephalosporins (ceftriaxone, cefotaxime, and ceftazidime) and monobactams (aztreonam) but not cephamycins or carbapenems. They are inhibited in vitro by clavulanate” [2]. Metallo-beta-lactamases (MBLs) are carbapenems hydrolyzing enzymes; inhibited by metal chelating agents like EDTA [3, 4].

Biofilms are the bacterial aggregates firmly lodged in the extracellular matrices of polysaccharides, proteins, enzymes, and nucleic acids; thereby, facilitating anchorage to any surfaces irreversibly [5, 6]. The matrix confers antibiotic resistance through processes such as expression of chromosomally encoded resistant genes, restriction of antibiotics, reduction in growth rate, and even counteracting the host immunity [5, 7, 8].

The biofilm formation and beta-lactamases production synergistically contribute for extensive dissemination of multi-drug resistant strains of gram-negative bacilli. They are responsible for implicating chronicity, persistence, and relapse of infections leading to high morbidity and mortality; thus, posing a serious health crisis [9, 10].

In this regard, the knowledge of biofilm formation and antibiogram of bacterial isolates is of utmost importance for rendering reliable empirical antibiotic therapy to the patients [11]. Irrespective of development of numerous molecular detection techniques, the conventional methods are economical and reliable for the routine screening and diagnosis [8, 12]. Hence, this study was conducted to detect the biofilm formation by gram-negative bacilli and determine their antimicrobial resistance pattern along with the detection of ESBL and MBL production.

## Main text

### Methods

This cross-sectional study was conducted at the Department of Microbiology in B.P. Koirala Institute of Health Sciences (March–June, 2018). 314 non-repetitive gram-negative isolates were recovered from the clinical specimens (blood, urine, pus, CSF, endotracheal tube, tracheal aspirate, fluids, lesion swab, genital swab, catheter tips, sputum) submitted to the microbiology lab for routine culture and sensitivity testing. The samples were received from various inpatients wards and outpatient departments of this hospital. All specimens were inoculated on Blood and MacConkey agar except urine specimens which were plated on Cysteine Lactose Deficient Medium (CLED) as per the standard bacteriological procedures. The culture plates were incubated at 35 °C for 24–48 h. The growth isolates were identified on the basis of colony morphology, pigmentation, odor, and their unique biochemical tests [1].

#### Antibiotic susceptibility test [13]

It was performed on Muller Hinton agar by Kirby Bauer disc diffusion method following CLSI guidelines. Ofloxacin (5 µg), levofloxacin (5 µg), ceftazidime (30 µg), cefepime (30 µg), amikacin (30 µg), gentamicin (10 µg), piperacillin (100 µg), piperacillin-tazobactam (100/10 µg), imipenem (10 µg), and colistin (10 µg) were tested as common antibiotics for all strains. Polymyxin B (300 units), tobramycin (10 µg) and carbenicillin (100 µg) were added for *Pseudomonas* spp.

#### Phenotypic detection of ESBL and MBL production [13, 14]

The ceftazidime resistant strains were screened for ESBL production by using disc diffusion test. The increase in the zone of the diameter of ≥ 5-mm between ceftazidime (30 μg) and ceftazidime-clavulanate (30/10 μg) was considered ESBL positive [14].

Whereas, imipenem-resistant strains were tested for MBL production by combined disc diffusion assay using two imipenem discs, one with added 10 µl of 0.5 M EDTA. The increased zone of inhibition of > 7 mm around the imipenem-EDTA disc in comparison to zone size of imipenem disc alone was confirmed positive for MBL production [14].

#### Biofilm detection by tube adherence and Congo red agar methods

##### Tube adherence method [15]

A growth organism was inoculated into trypticase soy broth and incubated for 24 h at 35 °C. After discarding the supernatant, the tube was washed with phosphate buffer saline. It was treated with 0.1% crystal violet for staining and then washed with water and dried. The appearance of visible biofilm lining the bottom and wall of the tube was considered positive.

##### Congo red agar method [16–18]

The organisms were inoculated on Congo red agar plate and incubated at 37 °C for 24 h. The formation of black colonies indicated biofilm production.

### Results

Out of 2562 samples received during the study period, only 314 were gram-negative bacilli showing the total growth rate of 12.25%. 314 isolates were distributed as *Escherichia coli* (38%), *Acinetobacter* spp. (20%), *Klebsiella* spp. (16%), *Pseudomonas* spp. (12%), *Enterobacter* spp. (9%), *Citrobacter* spp. (2%), *Proteus* spp. (2%), *Providencia stuartii* (1%), and *Salmonella Typhi* (1).

#### Antimicrobial resistant pattern (Table 1)

Among all the tested antibiotics, most strains were found to be highly resistant to ceftazidime whereas all strains were sensitive to colistin.

**Table 1:** Showing antibiotic resistance pattern

Antibiotics tested	*Escherichia coli* (121)	*Acinetobacter* spp. (63)	*Klebsiella* spp. (49)	*Pseudomonas* spp. (38)	*Enterobacter* spp. (27)	*Citrobacter* spp. (8)	*Proteus* spp. (5)	*Providencia stuartii* (2)
Amikacin	13.22%	44.44%	40.81%	34.21%	33.33%	12.5%	40%	0%
Gentamicin	23.97%	50.79%	46.94%	34.21%	48.14%	25%	60%	50%
Ofloxacin	61.98%	55.56%	57.14%	31.58%	37.03%	12.5%	60%	50%
Levofloxacin	51.24%	52.38%	55.1%	31.58%	29.63%	25%	60%	0%
Ceftazidime	61.98%	73.02%	75.51%	36.84%	51.85%	50%	40%	100%
Cefepime	52.07%	71.43%	59.18%	36.84%	44.44%	50%	40%	50%
Imipenem	21.49%	39.68%	53.01%	31.58%	37.03%	0%	60%	0%
Piperacillin	56.19%	55.56%	63.27%	31.58%	44.44%	62.5%	60%	0%
Piperacillin–Tazobactam	19.0%	46.03%	40.82%	21.01%	33.33%	25%	40%	0%
Colistin	0%	0%	0%	0%	0%	0%	N/A	N/A
Polymyxin B				10.53%				
Tobramycin				31.58%				
Carbenicillin				39.47%				

Not applicable (N/A) as they are intrinsically resistant

PolymyxinB, Tobramycin, and Carbenicillin were applied only for *Pseudomonas* spp.

#### Biofilm detection

A total of 197 (62.73%) isolates were biofilm positive as detected by either tube adherence or congo red agar method.

#### Phenotypic detection of ESBL, MBL and biofilm production (Table 2)

Majority of *Klebsiella* spp. (77.55%) were observed to be biofilm producers followed by *Pseudomonas* spp. (73.68%).

**Table 2:** Distribution of biofilm formers and ESBL and MBL producers on the basis of specific organisms

Organisms	Biofilm formers no. (%)	ESBL producers no. (%)	MBL producers no. (%)	Both ESBL and MBL producers no. (%)	ESBL, MBL and biofilm producers no. (%)
*Escherichia coli* (121)	73 (60.33%)	46 (38.01%)	11 (9.09%)	3 (2.48%)	3 (2.48%)
*Acinetobacter* spp. (63)	34 (53.97%)	10 (15.87%)	13 (20.63%)	1 (1.59%)	1 (1.59%)
*Klebsiella* spp. (49)	38 (77.55%)	15 (30.61%)	13 (26.53%)	3 (6.12%)	3 (6.12%)
*Pseudomonas* spp. (38)	28 (73.68%)	6 (15.79%)	10 (26.31%)	2 (5.26%)	2 (5.26%)
*Enterobacter* spp. (27)	16 (59.26%)	2 (7.41%)	3 (11.11%)	1 (3.70%)	0
*Citrobacter* spp. (8)	5 (62.50%)	2 (25%)	0	0	0
*Proteus* spp. (5)	2 (40%)	1 (20%)	1 (20%)	0	0
*Providencia stuartii* (2)	1 (50%)	2 (100%)	0	0	0
Total (314)	197 (62.73%)	84 (26.75%)	51 (16.24%)	10 (3.18%)	9 (2.87%)

Among the suspected cases of ESBL and MBL producers, 84 (26.75%) and 51 (16.24%) were confirmed as ESBL and MBL producers respectively. 38.01% of *E. coli* were detected as ESBL producers showing comparatively higher incidence. *Klebsiella* spp. were found to be the highest MBL producers i.e., 26.53% closely followed by *Pseudomonas* spp. (26.31%).

#### Association of ESBL and MBL production with biofilm formation

Although maximum ESBL producers were biofilm positive, no statistical significance was observed. (χ^2^ = 0.006, *P*-value= 0.937).

The association between MBL production and biofilm formation was found to be statistically significant. (χ^2^ = 10.20, *P*-value= 0.002).

#### Association of ESBL, MBL and Biofilm production with antibiotic resistance (Table 3)

There was a significant association of ESBL production and biofilm formation to antibiotic resistance. For all antibiotics except polymyxin B, the association between MBL production and antibiotic resistance was noted to be statistically significant (*P*-value = 0.001).

**Table 3:** Showing association between antibiotics resistance, ESBL production, MBL production, and biofilm production

Antibiotics	ESBL			MBL			Biofilm		
	Resistance (%)		*P*-value	Resistance (%)		*P*-value	Resistance (%)		*P*-value
	Producers (%)	Non-producers (%)		Producers (%)	Non-producers (%)		Formers (%)	Non-formers (%)	
Amikacin	22.61	30.43	0.174	84.31	17.49	0.001	34.52	17.95	0.002
Gentamicin	41.67	35.21	0.295	82.35	28.13	0.001	42.64	27.35	0.007
Ofloxacin	69.04	46.52	0.001	96.08	44.11	0.001	57.87	43.59	0.01
Levofloxacin	58.33	42.6	0.013	94.12	37.64	0.001	54.31	34.19	0.001
Cefepime	84.52	43.48	0.001	94.12	46.77	0.001	59.39	46.15	0.023
Ceftazidime	100	47.82	0.001	96.08	55.13	0.001	66.5	53.85	0.026
Imipenem	26.19	35.22	0.132	100	19.77	0.001	39.09	22.22	0.002
Piperacillin	72.62	45.65	0.001	90.19	45.62	0.001	57.87	44.44	0.021
Piperacillin-Tazobactum	35.71	27.39	0.153	66.67	22.43	0.001	32.49	24.79	0.148
Colistin	0	0	*	0	0	*	0	0	*
Polymyxin B	16.67	9.38	0.241	20	7.14	0.079	10.71	10	0.33
Tobramycin	66.67	25	0.067	80	14.28	0.001	35.71	20	0.233
Carbenicillin	66.67	34.38	0.126	80	25	0.001	42.86	30	0.267

PolymyxinB, Tobramycin, and Carbenicillin were applied only for *Pseudomonas* spp.

* No resistance was observed, P-value could not be calculated

### Discussion

In this study, 314 g-negative strains were obtained. *E. coli* was the most prevalent pathogen with the occurrence of 38%; identical to the results recorded by Fatima et al. (38%) [19]. Several studies reported *E. coli* to be the most isolated GNB causing septicemia, UTIs and other infections [19–22]. The incidences of *Acinetobacter* spp. (20%) and *Pseudomonas* spp. (12%) in our study coincide with the findings of Sundaram et al. (21%) and Mshana et al. (12.2%) respectively [21, 23].

On account of antibiotic resistance pattern, almost all organisms were highly resistant to ceftazidime. This is probably due to the widespread use of third-generation cephalosporins without knowing the severity of infections [23]. In contrast, no strains were resistant to colistin.

Overall, 62.73% of isolates were detected as biofilm formers in our study. This is in agreement with the study led by Allam et al. (64.28%) [24]. A similar study conducted in our center, BPKIHS, by Shrestha et al. recorded 71.8% isolates as biofilm producers [8]. The prevalence of biofilm producing *Klebsiella* spp. (77.55%), *Pseudomonas* spp. (73.68%), *E. coli* (60.33%) and *Acinetobacter* spp. (53.97%) in our study are in accordance with the studies of Allam et al. (72.72%), Pittaya et al. (79.4%), Allam et al. (55.77%) and Gurung et al. (50%) respectively [24–26]. The majority of biofilm producers were extracted from tissues, sputum, devices, and exudate samples supporting the fact that biofilm formation is facilitated by tissue lesions, implanted medical devices, chronic respiratory diseases, surgical wounds etc. [6, 9].

26.75% strains were accounted as ESBL producers coinciding with Mshana et al. (29.8%) [23]. The proportion of ESBL production in Nepal ranges from 18 to 62.7% [27]. Among the maximum isolated strains, the occurrence was higher for *E. coli* (38.01%) comparable to the result of Sundaram et al. (35.3%) [21]. The incidence of ESBL production by *Klebsiella* spp. in Nepal has been reported ranging from 4.1 to 90.9% supporting our finding [27, 28]. Laudy et al. had the result of 15% ESBL producing *P. aeruginosa* equals to our finding [29]. Moreover, 16.24% were MBL positive, concomitant with Kamalraj et al. (18%) [30]. Our study reported *Klebsiella* spp. (26.53%), *Pseudomonas* spp. (26.31%) and *Acinetobacter* spp. (20.63%) as the major MBL producers which are in conformity with the data of Kaur et al. (34.8%), Anuradha et al. (28.57%) and Baniya et al. (22%) for the respective organisms [18, 20, 31]. The coexistence of biofilm along with both beta-lactamases producing strains was found to be 2.87%. The biofilm matrix has been reported to enhance the expression of resistant genes like beta-lactamases [5, 7].

Despite the maximum ESBL producers being biofilm positive, no significant association was found between them, similar to the finding of Emami et al. but in contrast with other studies [17, 32, 33]. Conversely, the association between MBL production and biofilm formation was observed to be statistically significant. Although most of the antibiotics were resistant towards ESBL producers, the association was statistically insignificant. Amikacin followed by imipenem were found to be effective against ESBL producers. Except for polymyxin B, statistically significant association was established between MBL production and antibiotic resistance. Ceftazidime was 100% resistant to MBL positive strains, compatible with the study of Lyra et al. [34]. Besides, various studies have demonstrated MBL producers as pan drug-resistant strains signifying an alarming threat [35–37].

The association between biofilm and antibiotic resistance was noted to be statistically significant for aminoglycosides, fluoroquinolones, cephalosporins, imipenem, and piperacillin. A higher proportion of antibiotic resistance in biofilm producers in comparison to non-producers has been documented in many studies [35, 38–42]. No resistance was observed against colistin. It’s the last viable option for multi-drug resistant strains either being non-producers or producers of ESBL, MBL and biofilm [20, 43].

### Conclusion

The notable prevalence of biofilm-forming and multidrug-resistant organisms in our institution provides a glimpse of upcoming threat in our part of the world. The routine monitoring of biofilm and beta-lactamases production; therefore, can be recommended in clinical laboratories along with the strict implementation of infection control and prevention activities.

## Limitations

The lack of confirmation of biofilm, ESBL, and MBL production by using molecular technologies are the drawbacks of this study.

## Abbreviations

GNB: gram-negative bacilli
ESBL: extended-spectrum beta-lactamase
MBL: metallo-beta-lactamase
MDR: multidrug-resistant
UTI: urinary tract infection
CLSI: Clinical and Laboratory Standards Institute
EDTA: ethylene diamine tetra acetic acid
